# INVESTIGATING WALNUT CONSUMPTION AND COGNITIVE HEALTH IN A REPRESENTATIVE SAMPLE OF OLDER U.S. ADULTS

**DOI:** 10.1093/geroni/igz038.2441

**Published:** 2019-11-08

**Authors:** Nicholas J Bishop, Krystle E Zuniga

**Affiliations:** 1 Texas State University San Marcos, San Marcos, Texas, United States; 2 Texas State University, San Marcos, Texas, United States

## Abstract

Population aging increases the need to identify modifiable risk factors of cognitive decline such as nutritional intake. Several nutrients found in walnuts appear to play a neuro-protective role, yet few studies examine whole walnut consumption or draw from representative longitudinal samples. We draw observations from the nationally-representative Health and Retirement Study and Health Care and Nutrition Study to investigate the association between walnut consumption and cognitive trajectories among older US adults. The analytic sample consisted of 6,639 adults age 50 and over in 2013, representing a population of 77,726,682 community-dwelling older adults. Walnut consumption was a categorical measure representing no consumption, moderate consumption (< one serving per week), or high consumption (≥ one serving per week). Indicators of cognitive function representing working memory (immediate and delayed word recall) and global cognitive function (Telephone Interview of Cognitive Status, TICS) were measured at 3 time points (2012, 2014, and 2016). Latent growth models were used to estimate each linear trajectory while adjusting for covariates and complex survey design. Walnut consumption was positively associated with word recall and global mental status at baseline, but was not associated with change over the four year observational window. For example, those with high walnut consumption had baseline TICS scores .89 units greater (SE = .17, p < .001) than those consuming no walnuts. These results indicate that walnut consumption appears to have a positive association with cognitive health, but walnut consumption does not appear to be associated with short-term change in the cognitive outcomes measured.

